# MRI in head and neck cancer following chemoradiotherapy: what is the optimal delay to demonstrate maximal response?

**DOI:** 10.1007/s00330-021-07913-x

**Published:** 2021-05-19

**Authors:** S. E. J. Connor, C. Burd, N. Sivarasan, V. Goh

**Affiliations:** 1 School of Biomedical Engineering & Imaging Sciences Clinical Academic Group, King’s College London, London, UK; 2 Department of Radiology, Guy’s and St Thomas’ Hospital, London, UK; 3 Department of Neuroradiology, King’s College Hospital, London, UK; 4 Department of Radiology, Royal Brompton and Harefield NHS Foundation Trust, London, UK

**Keywords:** Diffusion magnetic resonance imaging, Squamous cell carcinoma of head and neck, Chemoradiotherapy, Treatment outcome, Human papillomavirus

## Abstract

**Objectives:**

To investigate the optimal timing for post-chemoradiotherapy (CRT) reference magnetic resonance imaging (MRI) in head and neck cancer, so as to demonstrate a maximal treatment response. To assess whether this differs in human papillomavirus–related oropharyngeal cancer (HPV-OPC) and whether the MRI timing impacts on the ability to predict treatment success.

**Methods:**

Following ethical approval and informed consent, 45 patients (40 male, mean age 59.7 ± 7.9 years, 33 HPV-OPC) with stage 3 and 4 HNSCC underwent pre-treatment, 6- and 12-week post-CRT MRIs in this prospective cohort study. Primary tumour (*n* = 39) size, T2w morphology and diffusion weight imaging (DWI) scores, together with nodal (*n* = 42) size and necrotic/cystic change, were recorded. Interval imaging changes were analysed for all patients and according to HPV-OPC status. MRI descriptors and their interval changes were also compared with 2-year progression-free survival (PFS).

**Results:**

All MRI descriptors significantly changed between pre-treatment and 6-week post-treatment MRI studies (*p* < .001). Primary tumour and nodal volume decreased between 6- and 12-week studies; however, interval changes in linear dimensions were only evident for HPV-OPC lymph nodes. Nodal necrosis scores also evolved after 6 weeks but other descriptors were stable. The 6-week nodal necrosis score and the 6- and 12-week nodal volume were predictive of 2-year PFS.

**Conclusion:**

Apart from HPV-OPC patients with nodal disease, the 6-week post-CRT MRI demonstrates maximal reduction in the linear dimensions of head and neck cancer; however, a later reference study should be considered if volumetric analysis is applied.

**Key Points:**

*• This study provides guidance on when early post-treatment imaging should be performed in head and neck cancer following chemoradiotherapy, in order to aid subsequent detection of recurrent tumour.*

*• Lymph nodes in HPV-related oropharyngeal cancer patients clearly reduced in size from 6 to 12 weeks post-treatment. However, other lymph node disease and all primary tumours showed only a minor reduction in size beyond 6 weeks, and this required a detailed volumetric analysis for demonstration.*

*• Timing of the reference MRI following chemoradiotherapy for head and neck cancer depends on whether the patient has HPV-related oropharyngeal cancer and whether there is nodal disease. MRI as early as 6 weeks post-treatment may be performed unless volumetric analysis is routinely performed.*

## Introduction

Chemoradiotherapy (CRT) is the principal treatment option for stage 3 and 4 head and neck squamous cell carcinoma (HNSCC). Despite curative intent, post-treatment loco-regional failure has been shown to occur in more than 25% of such patients [1, 2]. Recurrent disease may be managed with salvage surgery; however, this is ideally performed at an early stage before the onset of fibrosis and before it becomes irresectable.

The detection of residual or recurrent HNSCC by clinical examination maybe challenging due to post-treatment changes, whilst biopsies may be unreliable and add to morbidity [3–6]. 18F-Fluorodeoxygluocose (^18^F-FDG) positron emission tomography (PET) [7], quantitative diffusion-weighted (DW) MRI [8–12] and qualitative MRI descriptors have all been used to aid tumour detection in the post-treatment setting. MRI descriptors such as T2w and DWI signal, morphology and dimensions have been demonstrated to contribute to both the early post-treatment and later symptomatic assessment of recurrent disease [13–23].

MRI may be used as reference imaging to help evaluate for future recurrence, whilst it can also provide important predictive information regarding the eventual treatment outcome at primary and nodal sites [16–20]. There is currently no data available on the evolution of the MRI findings or the optimal timing of MRI in the early post-treatment period. It would be useful to ascertain the earliest point at which the successfully treated tumour demonstrates the greatest response on imaging. This would allow for earlier post-treatment reference imaging and potentially earlier detection of recurrent tumour during imaging surveillance. In addition, the impact of human papillomavirus oropharyngeal cancer (HPV-OPC) status on the timing of post-treatment change should be explored since it is a potential confounding factor, with its differing morphological features and improved clinical outcomes [24, 25].

It was hypothesised that a 6-week post-CRT MRI in stage 3 and 4 HNSCC patients would be an appropriate reference MRI, since the maximal post-treatment response and prognostic imaging indicators will be observed. Thus, our primary objective was to determine whether there was an evolution in specific dimensions, morphology and signal of the primary tumour and largest lymph node between 6- and 12-week post-CRT MRI studies, and whether this was influenced by the HPV-OPC status. Secondary objectives were to investigate whether MRI features or their interval changes at these post-treatment time points were predictive of 2-year progression-free survival (PFS).

## Methods

### Participants

Participants were recruited for a prospective single-centre cohort observational study (http://www.controlled-trials.com/ISRCTN58327080) following Research Ethics Committee approval (REC reference 13/LO/1876) and informed consent.

Patients were eligible for the study if there was histologically confirmed stage 3 or 4 primary HNSCC without distant metastatic disease and a 1-cm^2^ area of measurable primary tumour and/or nodal tumour on the basis of standard clinico-radiological staging, and curative primary (chemo)radiotherapy was planned. Exclusion criteria were prior chemoradiotherapy, an ECOG performance status > 2, known allergy to gadolinium-based contrast medium or eGFR < 30 mL/min.

### HPV status

Biopsies were obtained from the primary or nodal site. HPV status was analysed for all oropharyngeal cancers and some other cancer sites. HPV testing comprised p16 using an immune-stain or for high-risk HPV DNA using in situ hybridisation.

### Treatment

Intensity-modulated radiotherapy (IMRT) was delivered as per the standard of care which was 70 Gy in 35 fractions, 2 Gy per fraction delivered once daily, 5 days a week. Concomitant intravenous cisplatin at a dose of 35 mg/m^2^ every 7 days, starting on day 1 of radiotherapy, was used for all patients with adequate GFR and no other contraindications, with carboplatin being used if measured GFR < 50 or patient had a history of hearing impairment.

### Imaging

Patients underwent MRI pre-treatment and at 6 and 12 weeks after the completion of CRT. MRI was performed with a 1.5-T system (Magnetom Aera, Siemen Healthcare GmbH) using a surface phased array neck coil. The MRI protocol and sequence parameters are listed in Table 1.

**Table 1:** MRI protocol

	Plane	Slice thickness/gap	TR/TE	Field of view	Number of averages	Pixel bandwidth	Flip angle	Acquisition matrix
T1w	Axial	4/0	549/11	220 × 220	1	200	160	384 × 269
T2w	Axial	4/0	5830/102	220 × 220	1	190	150	384 × 346
T1w fat-saturated-DIXON post-gadolinium	Axial	4/0	566/11	220 × 220	1	330	145	320 × 224
STIR	Coronal	3/0.3	3000/35 TI 140	260 × 260	1	220	160	320 × 224
T1w fat-sat-DIXON post-gadolinium	Coronal	3/0.3	708/10	280 × 280	1	340	145	320 × 320
Diffusion-weighted imaging	Axial (two slabs)	4/0.5	5900/60 *b* values 0, 50, 100, 800, 1500 s/mm^2^	240 × 240	2	1375	90	130 × 130

### Image analysis

The location of any measurable tumour (> 1 cm^2^) and largest measurable lymph node (> 1 cm^2^) was recorded at the time of entry into the study by a radiologist (24 years of experience). Two readers (4 and 5 years of experience) independently assessed the measurable pre-treatment followed by the 6- and 12-week post-treatment MRIs. Five test cases were evaluated prior to the study group to attain consistency on the scoring system. The observers were blinded to clinical information.

The post-gadolinium fat-saturated T1 axial sequence was primarily used for the delineation of primary tumour and the largest lymph node, but with access to the other sequences. Areas of peri-tumoural inflammation characterised by high T2w signal, free diffusion and avid gadolinium enhancement were not included. Standardised window widths were applied.

**Fig. 1:**
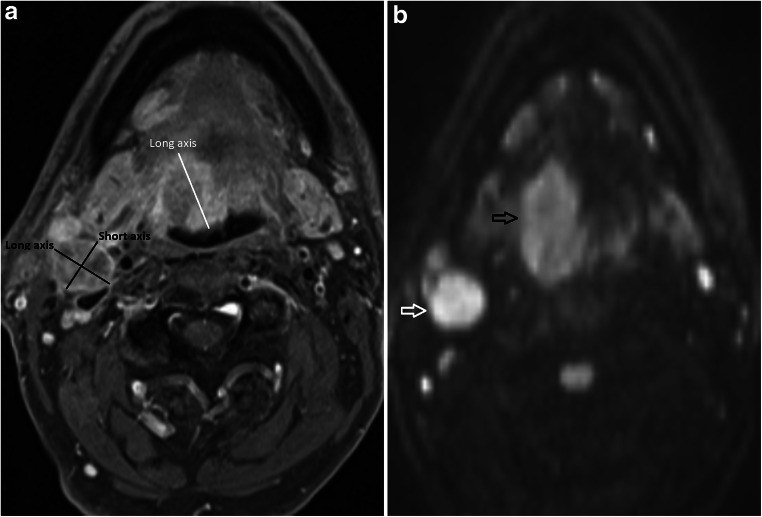
Measurable right base of tongue carcinoma and largest right level 2 lymph node to demonstrate dimensions evaluated. **a** Axial fat-saturated post-gadolinium T1w image demonstrates the long axial primary tumour measurement (white line), and both the long and short axial largest lymph node measurements (black lines) are depicted. There is necrotic/cystic change recorded in the largest lymph node. **b** DWI *b* = 800 axial image is used to aid the delineation of tumour. The highest DWI signal of the tumour (black open arrow) was recorded as 2 (isointense to cord) and that of the lymph node (white open arrow) was recorded as 3 (moderately hyperintense to cord)

**Fig. 2:**
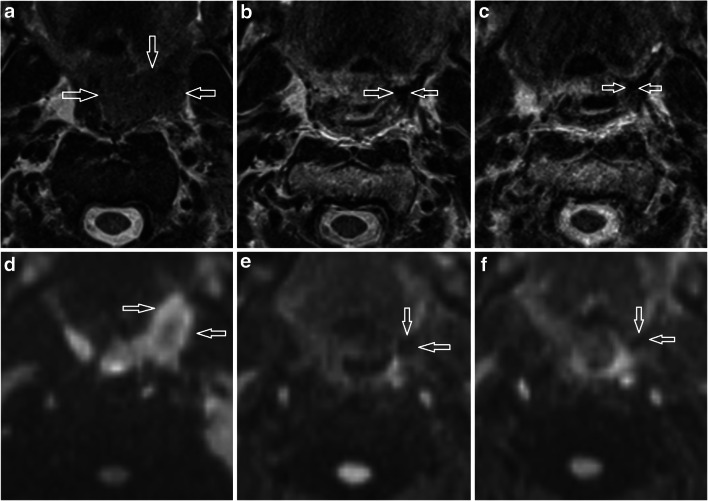
Left palatine tonsillar carcinoma to illustrate T2w morphology and DWI scoring. **a**–**c** T2w axial images on **a** pre-treatment, **b** 6-week post-treatment and **c** 12-week post-treatment MRI. The T2 morphology scores (lesions indicated by open white arrows) were 2 on pre-treatment, 0 on 6-week post-treatment and 0 on 12-week post-treatment MRIs. **d**–**f** DWI *b* = 800 axial images on (**d**) pre-treatment, (**e**) 6-week post-treatment and (**f**) 12-week post-treatment MRIs. The DWI scores (lesion indicated by open white arrows) were 3 on pre-treatment, 1 on 6-week post-treatment and 1 on 12-week post-treatment MRIs

**Fig. 3:**
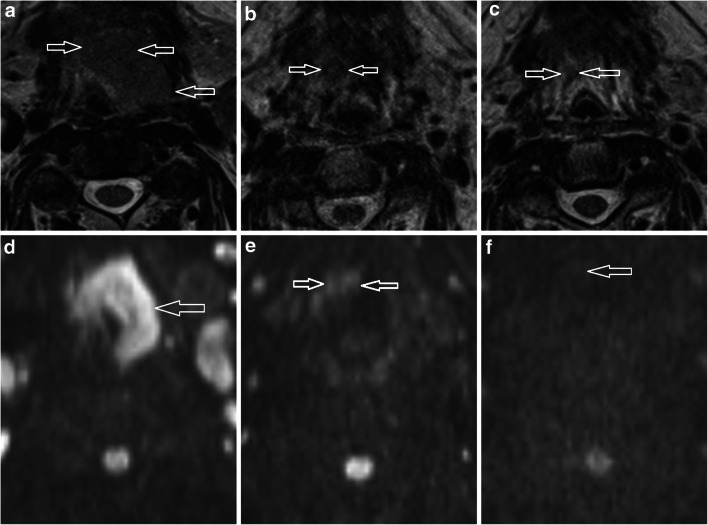
Bilateral base of tongue carcinoma more marked on the left to illustrate T2w morphology and DWI scoring. **a**–**c** T2w axial images on (**a**) pre-treatment, (**b**) 6-week post-treatment and (**c**) 12-week post-treatment MRI. The T2 morphology scores (lesions indicated by open white arrows) were 2 on pre-treatment, 1 on 6-week post-treatment and 1 on 12-week post-treatment MRIs. **d**–**f** DWI *b* = 800 axial images on **d** pre-treatment, **e** 6-week post-treatment and **f** 12-week post-treatment MRIs. The DWI scores (lesions indicated by open white arrows) were 4 on pre-treatment, 1 on 6-week post-treatment and 0 on 12-week post-treatment MRIs

The primary tumour long axial dimension and volume were measured (Fig. 1). The primary tumour T2w morphological score was adapted from a previously described scale [16]: 0, no visible mass lesion; 1, uniformly low T2w signal lesion with flat, retracted margins; 2, mass with characteristics not defined by grade 1 or 3; 3, intermediate T2w signal mass ≥ 10 mm with expansile margins (Figs. 2 and 3). The primary tumour maximal DWI signal (*b* = 800) was categorised as follows: 0, no visible mass on DWI; 1, hypointense to cord; 2, isointense to cord; 3, moderately hyperintense to cord; and 4, significantly hyperintense to cord (Figs. 1, 2 and 3).

The largest measurable lymph node short- and long-axis dimensions and volume were measured (Fig. 1). The largest lymph node was also assessed for the presence of central areas of non-enhancement on the post-gadolinium fat-saturated T1 axial sequence consistent with cystic/necrotic features. This was scored as 0, no necrosis; 1, < 50% necrosis/cystic change and 2, > 50% necrosis/cystic change.

If the observers’ measurements or scores differed, then consensus was achieved with input from a third radiologist (24 years’ experience). The third radiologist also measured the volume (including solid enhancing and necrotic components) of the primary tumour and the largest lymph node on the pre-treatment and 6- and 12-week post-treatment MRI studies. This was evaluated with a summation of areas technique.

### Clinical follow-up

Clinical assessment was performed at 1 year and 2 years following completion of chemoradiotherapy. The outcome of a 12-week 18F-FDG PET/computed tomography (18F-FDG PET/CT) study was initially used to guide management as was the standard of care. Recurrent loco-regional and systemic disease was determined by cytological or histological confirmation or by serial progression on imaging follow-up. Two-year PFS was recorded according to whether there was any sign of cancer by 2 years following completion of CRT.

### Statistical analysis

Inter-observer agreement was calculated with interclass correlation (ICC) for primary tumour and nodal linear dimensions and Cohen’s kappa for categorical scores.

Consensus score values and the mean of the two recorded tumour linear dimension measures were used for further analysis.

Changes in the primary tumour and nodal MRI features between pre-treatment to 6 weeks post-treatment, and 6 to 12 weeks post-treatment MRI studies were analysed. These comparisons were performed for all cases and then separately for both HPV-OPC and other HNSCC.

The primary tumour and nodal MRI features at 6 and 12 weeks, as well as the changes between pre-treatment to 6 weeks and pre-treatment to 12 weeks, were compared with 2-year PFS.

Statistical analysis was performed using Microsoft Excel. All tests were two tailed and a *p* value of < 0.05 was considered significant for the comparison of interval changes between primary tumour and nodal MRI features and for the comparison of MRI features and their interval changes with 2-year outcomes.

For continuous data, paired *t* tests were applied when normally distributed according to the Kolmorogov-Smirnov test, whereas the Mann-Whitney *U* test was applied when it was not normally distributed. Chi-squared test was used to analyse categorical scores.

## Results

### Descriptive statistics

**Fig. 4:**
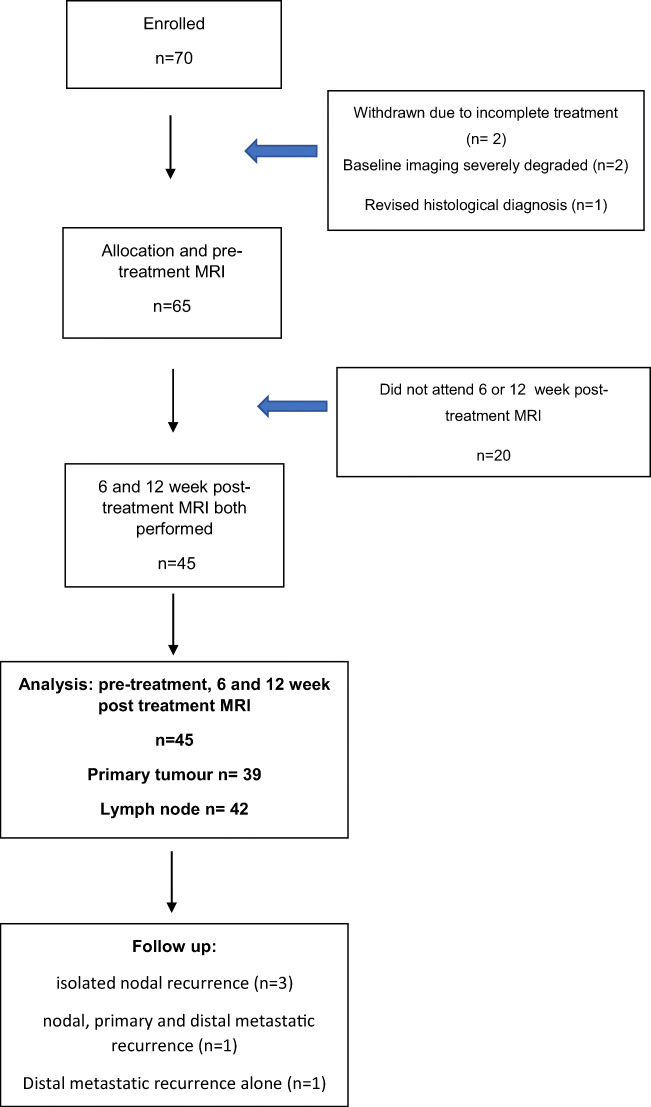
Participant flow-chart

The participant flowchart is summarised in Fig. 4. There were 70 patients initially enrolled in the study. Patients were subsequently withdrawn from the study (*n* = 5) or did not attend for either the 6- or 12-week post-treatment MRIs (*n* = 20).

There were 45 patients analysed (40 male, 5 female, mean age 59.7 ± 7.9 years). The tumour site, subsite and HPV-OPC status are documented in Table 2. There were 33 patients with HPV-OPC and 12 with other HNSCC. Measurable tumour was delineated at the primary site alone (*n* = 3), the largest lymph node site (*n* = 6) or both sites (*n* = 36), so there were 39 patients with measurable primary tumour and 42 patients with measurable nodal disease. There were 37 patients with stage 4 (82%) and 8 patients with stage 3 (18%) disease. The primary site, nodal staging and HPV status are demonstrated in Table 1. Cisplatin was administered in 38 patients and carboplatin in 7 patients. At 2-year follow-up, there were 5/45 patients with disease progression by 2 years (Fig. 4). Nodal recurrence always occurred at the site of the largest lymph node analysed.

**Table 2:** Primary subsite, TN staging and HPV status of the 45 patients

	Subsite			T stage					N stage					HPV status		
				T0	T1	T2	T3	T4	N0	N1	N2A	N2B	N2C	+ve	−ve	Not tested
Oropharynx (*n* = 35)	Tongue base (17)	Tonsil (17)	Soft palate (1)	0	6	9	7	13	3	4	3	21	4	33	2	0
Larynx (*n* = 5)	Supra glottic (4)	Trans glottic (1)					4	1	0	2	2	1	0	0	1	4
Hypopharynx (*n* = 5)	Piriform fossa (5)					3	1	1				4	1	0	0	5

The ICCs for primary tumour and nodal linear dimensions were 0.9–0.95, 0.79–0.88 and 0.82–0.86 for pre-treatment, and 6- and 12-week post-treatment MRIs. The Cohen’s kappa statistics for the qualitative scores were 0.8–1, 0.85–1 and 0.87–1 on the pre-treatment, and 6- and 12-week post-treatment MRIs.

### Evolution of 6- and 12-week post-treatment MRI descriptors

The primary tumour (long axial and volume) dimension, DWI signal and T2w morphological scores as well as nodal (long/short axial linear and volume) dimensions and presence of necrotic/cystic signal at each timepoint are demonstrated in Table 3. The interval changes between pre-treatment, 6- and 12-week MRIs and *p* values for the statistical differences are also recorded in Table 3. The separate analyses for HPV-OPC and other HNSCC patients are shown in Table 4.

**Table 3:** Dimensions and qualitative measures of primary tumour and lymph node on 0-, 6- and 12-week MRI studies with interval changes for all patients

Primary tumour							
	Pre (*n* = 39)	6 (*n* = 39)	12 (*n* = 39)	0–6 absolute and % change (*n* = 39)	*p* value	6–12 absolute and % change (*n* = 39)	*p* value
Linear long axis (mm)	27 [21.5, 31.5]	8 [0, 13]	8 [0, 13]	19 [14, 25.5] − 73.3% [− 100, − 54.6%]	***p < .001***	0 [0, 0] 0 [0, 0]	*p* = .98
Volume (mm^2^)	10,506 [5304, 13,446]	562 [64, 1068]	480 [0, 908]	9908 [3672, 12,426] − 95.5% [− 89.6, − 99.4%]	***p < .001***	36 [16,82] − 6.9% [− 2.6, − 14.6%]	***p < .001***
T2w morphology score 0	0	19	19				
T2w morphology score 1	0	11	11				
T2w morphology score 2	0	9	9				
T2w morphology score 3	39	0	0				
T2w morphology mean score	3	0.7	0.7		***p < .001***		*p* = 1
DWI score 0	0	19	19				
DWI score 1	2	20	20				
DWI score 2	8	0	0				
DWI score 3	9	0	0				
DWI score 4	20	0	0				
DWI mean score	3.2	0.5	0.5		***p < .001***		*p* ***=*** 1
Lymph node							
	Pre (*n* = 42)	6 (*n* = 42)	12 (*n* = 42)	0–6 absolute and % change (*n* = 42)	*p* value	6–12 absolute and % change (*n* = 42)	*p* value
Linear long axis (mm)	26 [19.5, 32.8]	12 [10, 16]	10 [9, 13]	12 [6.3, 18.8]− 50.8% [− 59.6, − 34.3%]	***p < .001***	2 [0, 3] − 12.9% [− 23.2, 0%]	***p < 0.05***
Linear short axis (mm)	18.5 [14, 22.8]	8.5 [6, 10.8]	7 [6, 9]	9.5 [7,14]− 56.6% [− 64.8, − 42.5%]	***p < .001***	1.5 [0, 2] − 16.7% [− 24.3, 0%]	***p < 0.05***
Volume (mm^2^)	4902 [2717, 10,161]	852 [463, 1232]	496 [246, 849]	3363 [1818, 9243] − 81.5% [− 68.8, − 92.0%]	***p < .001***	235 [108, 443] − 31.5% [− 13.5, − 46.4%]	***p < .001***
Necrosis score 0	6	22	30				
Necrosis score 1	26	18	9				
Necrosis score 2	10	2	3				
Necrosis mean score	1.1	0.5	0.4		***p < .001***		***p < 0.05***

*p* < 0.05 or *p* < 0.001 are statistically significant (marked in bold)

Summary statistics: median [inter-quartile range]; mean ± standard deviation

**Table 4:** Dimensions and qualitative measures of primary tumour and lymph node on 0-, 6- and 12-week MRI studies with interval changes for HPV-OPC and other HNSCC participants

Primary site HPV-OPC							
	Pre (*n* = 29)	6 (*n* = 29)	12 (*n* = 29)	0–6 absolute and % change (*n* = 29)	*p* value	6–12 absolute and % change (*n* = 29)	*p* value
Long axis (mm)	27 [21, 34]	0 [0, 14]	0 [0, 14]	18 [14, 27] − 100% [− 100, − 47.4%]	***p < .001***	0 [0, 0] 0 [0, 0]	*p* = .98
Volume (mm^2^)	10,834 [6312, 17,199]	392 [75, 922]	360 [40, 887]	10,291 [5985, 14,251] − 96.5% [− 92.5, − 99%]	***p < .001***	36 [20.5, 66.5] − 7.5% [− 3.2, − 17.7%]	***p < .001***
T2w morphology score 0	0	15	15				
T2w morphology score 1	0	9	9				
T2w morphology score 2	0	5	5				
T2w morphology score 3	29	0	0				
T2w morphology mean score	2	0.7	0.7		***p < .001***		*p* ***= 1***
DWI score 0	0	15	15				
DWI score 1	1	14	14				
DWI score 2	5	0	0				
DWI score 3	6	0	0				
DWI score 4	17	0	0				
DWI mean score	3.3	0.5	0.5		***p < .001***		*p = 1*
Nodal site HPV-OPC							
	Pre (*n* = 32)	6 (*n* = 32)	12 (*n* = 32)	0–6 absolute and % change (*n* = 32)	*p* value	6–12 absolute and % change (*n* = 32)	*p* value
Long axis (mm)	27.5 [22.8, 33.3]	13 [10, 16.3]	9.5 [9, 13]	13.5 [8.8, 21] − 52.0% [− 60.2, 45.0%]	***p < .001***	1.5 [0.8, 3.3] − 11.8% [− 22.6, − 4.7%]	***p < .001***
Short axis (mm)	20.1 ± 7.2	8.4 ± 2.8	7.0 ± 2.2	11.6 ± 6.0 − 55.7% ± 14.1%	***p < .001***	1.5 ± 1.6 − 15.5% ± 17.0%	***p < .001***
Volume (mm^2^)	4902 [2820, 10,193]	894 [601, 1248]	540 [291, 838]	3363 [2187, 9303] − 81.5% [− 70.2, − 92.0%]	***p < .001***	275 [128, 465] − 32.9 [− 16.3, − 41.8%]	***p < .001***
Necrosis score 0	4	16	22				
Necrosis score 1	21	15	8				
Necrosis score 2	7	1	2				
Necrosis mean score	1.1	0.5	0.4		***p < .001***		***p < .001***
Primary site other HNSCC							
	Pre (*n* = 10)	6 (*n* = 10)	12 (*n* = 10)	0–6 absolute and % change (*n* = 10)	*p* value	6–12 absolute and % change (*n* = 10)	*p* value
Long axis (mm)	25 [22.8, 27.8]	8.5 [0, 9]	8.5 [0, 9]	21.5 [18.3, 24.3] − 70.60% [− 100, − 62.5%]	***p < .001***	0 [0, 0] 0 [0, 0]	*p = .97*
Volume (mm^2^)	8560 [3340, 12,360]	762 [0,1320]	654 [0, 1288]	8560 [2108, 11,164] − 93.3% [− 80.2, − 100%]	***p < .05***	36 [0,108] − 3.1% [0, − 14.7%]	***p < .05***
T2w morphology score 0	0	4	4				
T2w morphology score 1	0	3	3				
T2w morphology score 2	0	3	3				
T2w morphology score 3	10	0	0				
T2w morphology mean score	3	0.9	0.9		***p < .001***		*p = 1*
DWI score 0	0	4	4				
DWI score 1	1	6	6				
DWI score 2	3	0	0				
DWI score 3	4	0	0				
DWI score 4	2	0	0				
DWI mean score	2.7	0.6	0.6		***p < .001***		*p = 1*
Nodal site other HNSCC							
	Pre (*n* = 10)	6 (*n* = 10)	12 (*n* = 10)	0–6 absolute and % change (*n* = 10)	*p* value	6–12 absolute and % change (*n* = 10)	*p* value
Long axis (mm)	21.7 ± 8.4	12.3 ± 4.6	9.9 ± 4.2	9.4 ± 8.0 − 38.7% ± 21.3%	***p < .001***	2.4 ± 4.0 − 15.8% ± 32.6%	*p = .09*
Short axis (mm)	15.9 ± 4.9	7.7 ± 2.2	7.1 ± 2.0	8.2 ± 5.2 − 47.2% ± 19.8%	***p < .001***	0.6 ± 1.5 − 4.9% ± 21.9%	*p = .24*
Volume (mm^2^)	4649 [2008, 8701]	545 [254, 991]	357 [168, 908]	2683 [1561, 8453] − 83.7% [− 55.4, − 93.2%]	***p < .05***	120 [15.0, 385] − 19.9% [5.7, − 57.2%]	***p < .05***
Necrosis score 0	2	6	8				
Necrosis score 1	5	3	1				
Necrosis score 2	3	1	1				
Necrosis mean score	1.1	0.5	0.3		***p < .001***		***p < 0.05***

*p* < 0.05 or *p* < 0.001 are statistically significant (marked in bold)

Summary statistics: mean ± standard deviation or median [inter-quartile range]

There was a significant change in the primary tumour (linear and volume) dimensions, DWI signal and T2w morphological scores between pre-treatment and 6 weeks (*p* < 0.001). Only the primary tumour volume dimensions (− 6.9%) showed a significant change between 6 and 12 weeks, with stable interval DWI signal and T2w morphological scores and no significant reduction in the linear long axial dimension.

There was a significant reduction in nodal volume between both pre-treatment to 6-week and 6- to 12-week studies for all patients (*p* < 0.001) and regardless of HPV-OPC status. The nodal long/short axial dimensions only decreased further between the 6- and 12-week MRIs in the HPV-OPC lymph nodes (*p* < 0.001) with a non-significant reduction for other HNSCC. There were interval changes in nodal necrosis scores between pre-treatment to 6-week and 6- to 12-week studies irrespective of HPV-OPC status.

### Comparison of 6- and 12-week post-treatment MRI descriptors with 2-year outcomes

**Fig. 5:**
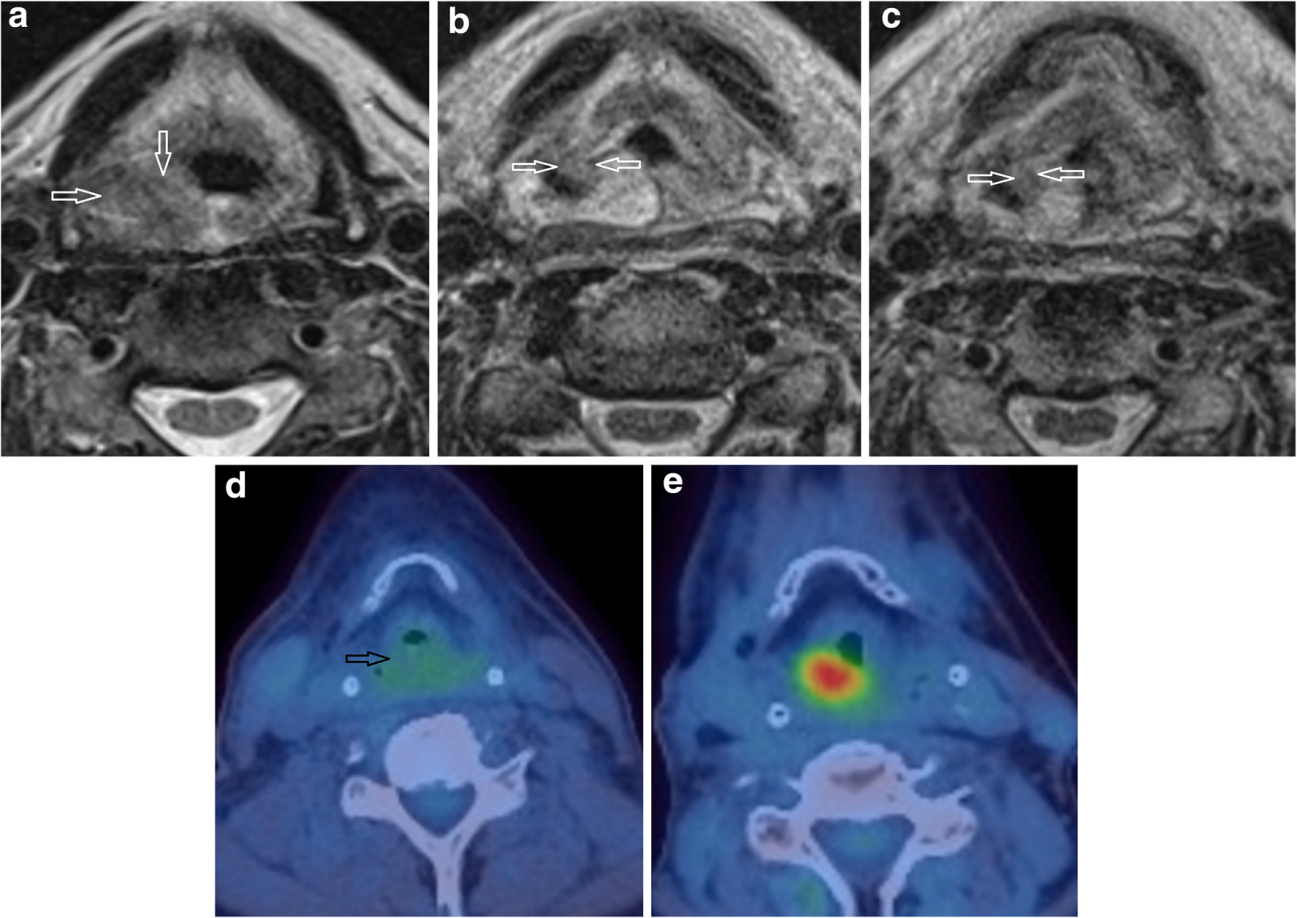
Right piriform carcinoma which recurred at 2-year follow-up. **a**–**c** T2w axial images on (**a**) pre-treatment, (**b**) 6-week post-treatment and (**c**) 12-week post-treatment MRI. The T2 morphology scores (lesions indicated by open white arrows) were 2 on pre-treatment, 1 on 6-week post-treatment and 1 on 12-week post-treatment MRIs. **d** 18-FDG PET-CT at 12 weeks post-treatment did not reveal any focal uptake but **e** subsequent 18-FDG PET -CT demonstrates focal uptake with a time to recurrence of 215 days

The primary tumour and nodal MRI descriptors on pre-treatment and 6- and 12-week MRI studies and their interval changes are compared between patients with and without disease progression at 2 years (Fig. 5) in Table 5. The 6-week nodal necrosis score and both the 6-week and 12-week nodal volume predicted 2-year PFS (*p* < 0.05). There was also a trend to a significant association between absolute primary tumour volume reduction at 6 weeks (*p* = 0.06) and 12 weeks (*p* = 0.06) with the 2-year PFS. There was no other correlation between any of the MRI descriptors on either 6- or 12-week post-treatment imaging or their interval changes, with the 2-year PFS (*p* = 0.22-1).

**Table 5:** Comparison of dimensions and qualitative measures of primary tumour and lymph node in patients with and without progression-free survival at 2 years

Primary tumour		Disease progression free (*n* = 34)	Disease recurrence (*n* = 5)	*p* value
6 weeks	Long axis (mm)	7.1 ± 7.5	6.2 ± 9.7	*p = .81*
	Volume (mm^2^)	509 [67, 1032]	720 [0, 2884]	*p = .87*
	T2w morphology score 0	16	3	
	T2w morphology score 1	10	1	
	T2w morphology score 2	8	1	
	T2w morphology score 3	0	0	
	T2w morphology mean score	0.8	0.6	*p = .70*
	DWI score 0	16	3	
	DWI score 1	18	2	
	DWI score 2	0	0	
	DWI score 3	0	0	
	DWI score 4	0	0	
	DWI mean score	0.5	0.4	*p = 1*
12 weeks	Long axis (mm)	7.2 ± 7.6	6.2 ± 9.65	*p = .80*
	Volume (mm^2^)	446 [30, 920]	620 [0, 2694]	*p = .74*
	T2w morphology score 0	16	3	
	T2w morphology score 1	10	1	
	T2w morphology score 2	8	1	
	T2w morphology score 3	0	0	
	T2w morphology mean score	0.8	0.6	*p = .70*
	DWI score 0	16	3	
	DWI score 1	18	2	
	DWI score 2	0	0	
	DWI score 3	0	0	
	DWI score 4	0	0	
	DWI mean score	0.5	0.4	*p = 1*
0–6 weeks change	Absolute tumour size (mm) and % tumour size reduction	20 [16, 26.8] − 70.6% [− 100, − 52.8%]	14 [14, 25] − 100% [− 100, − 59.1%]	*p = .46*
	Absolute volume (mm^2^) and % volume reduction	10,173 [7483, 12,764] − 96% [− 91.8, − 99.1%]	3268 [1260, 9266] 83.6% [70.7, 100]	*p = .06 p = .56*
0–12 weeks change	Absolute tumour size (mm) and % tumour size reduction	20 [16, 26.8] − 70.6% [− 100, − 52.8%]	14 [14, 25] − 100% [− 100, − 59.1%]	*p = .49*
	Absolute volume (mm^2^) and % volume reduction	10,473 [7490, 12,837] − 96.4% [− 92.5, − 99.9%]	3336 [1260, 9472] 85.9% [72.1, 100%]	*p = .06 p = .42*
Lymph node		Disease progression free (*n* = 37)	Disease recurrence (*n* = 5)	*p* value
6 weeks	Long axis (mm)	12 [9, 16]	14 [12, 16]	*p = .47*
	Short axis (mm)	8.1 ± 2.7	9.4 ± 1.1	*p = .31*
	Volume (mm^2^)	876 [446, 1180]	780 [409, 1634]	***p < .05***
	Necrosis score 0	18	4	
	Necrosis score 1	18	0	
	Necrosis score 2	1	1	
	Necrosis mean score	0.54 ± 0.56	0.4 ± 0.89	***p < .05***
12 weeks	Long axis (mm)	10 [9, 13]	9 [9, 10]	*p = .58*
	Short axis (mm)	7 [5, 9]	7 [7, 7]	*p = .70*
	Volume (mm^2^)	468 [244, 833]	696 [303, 1021]	***p < .05***
	Necrosis score 0	26	4	
	Necrosis score 1	8	0	
	Necrosis score 2	3	1	
	Necrosis mean score	0.39 ± 0.64	0.2 ± 0.48	*p = .14*
0–6 weeks change	Absolute long axis (mm) and % change	12 [7, 19] − 51.6% [− 59.6, − 37.0%]	13 [6, 18] − 48.1% [− 52.9, − 33.3%]	*p = .92 p = .47*
	Absolute short axis (mm) and % change	9 [7, 14] − 56% [− 65.4, − 43.8%]	13 [7, 14] − 59.1% [− 60.9, − 41.2%]	*p = .90*
	Absolute volume (mm^2^) and % change	3276 [1642, 9273] 80.8% [64.3, 92%]	4946 [4004, 10,204] 86.7% [78.5, 93.3%]	*p = .25*
0–12 weeks change	Absolute long axis (mm) and % change	13 [8, 20] − 58.1% [− 65.4, − 48.1%]	18 [16, 21] − 66.7% [− 69.6, − 61.8%]	*p = .38*
	Absolute short axis (mm) and % change	11 [7, 15] − 63.6% [− 72.5, − 55%]	15 [10, 15] − 68.2% [− 68.2, − 58.8%]	*p = .51*
	Absolute volume (mm^2^) and % change	3486 [1775, 9781] 89% [73, 95.1%]	5010 [4167, 10,770] 92% [81.2, 96%]	*p = .25 p = .55*

*p* < 0.05 or *p* < 0.001 are statistically significant (marked in bold)

Summary statistics are mean ± standard deviation or median [inter-quartile range]

Some caution should be exercised when interpreting the comparison with 2-year outcome due to the small number of participants (5/45) with tumour recurrence at 2 years.

## Discussion

All the proposed MRI descriptors changed significantly from pre-treatment to 6-week post-treatment studies. Whilst primary tumour and nodal volume dimensions continued to decrease between 6- and 12-week post-treatment MRIs, a significant change in linear dimensions was only demonstrated in HPV-OPC lymph nodes. Nodal necrosis reduced after 6 weeks; however, the other morphological and signal scores remained stable. The nodal necrosis score, absolute primary tumour volume reduction and nodal tumour volume showed prognostic potential at 6 weeks; however, there was only a small sample with recurrent disease so these results should be interpreted with caution.

A comparison with a post-treatment reference MRI aids the accurate interpretation of future follow-up imaging and the detection of recurrent disease. In order to optimise the identification of primary tumour or nodal progression, the imaging appearances should be compared with those at the time of greatest response. The demonstration of clear continued reduction in lymph node linear dimensions from 6 to 12 weeks post-treatment in HPV-OPC patients indicates that a later reference MRI would certainly be required in such cases. HPV-OPC status is of importance since it has unique histopathological characteristics, distinct epidemiology and improved response to CRT. Differing patterns of lymph node response have been noted in the HPV-OPC population, with a greater initial involution but then a more prolonged and inconsistent reduction in size, particularly in the presence of low-density lymph nodes on CT [24–26].

Whilst there have been a number of studies addressing the value of quantitative DW-MRI in the early post-CRT period for the prediction of residual disease, there is limited data on the value of primary tumour and nodal qualitative MRI descriptors and dimensions in predicting loco-regional or distant treatment failure [16, 27, 28]. Our choice of the qualitative MRI descriptors to evaluate was informed by previous studies exploring the prognostic significance of post-treatment MRI signal, morphological characteristics and size of residual masses, either alone or in combination. Our T2w signal and morphological criteria for the primary tumour evaluation were adapted from a study by King et al, which showed that a mass of low T2w signal and a flat-edged/retracted margin predicted treatment success, whereas a mass of intermediate T2w signal and > 1 cm with expansile margins was associated with treatment failure [16]. Other authors have included a combination of signal and enhancement characteristics in their evaluation of residual primary tumour for the purposes of predicting outcome [27, 28]. There have been mixed outcomes in studies investigating the prognostic value of nodal signal and morphology in the early post-treatment period [11, 16, 20]. Nodal ill definition, low T2w signal and necrosis were shown to be poorly predictive of treatment failure [18, 20], whilst one study showed increased DWI signal to be more specific for residual tumour than 18F-FDG PET/CT [11]. Our finding of 6-week necrosis score being predictive of 2-year PFS is of uncertain significance, due to the small sample of survivors and the likely influence of the prognostically favourable HPV-OPC cystic lymph nodes.

Tumour dimensions and their interval changes are key to the evaluation of post-treatment reference imaging, and have previously provided prognostic stratification both at primary tumour [29–31] and nodal [18, 33, 34] locations. Comparison of interval changes in absolute primary tumour volume at 6 and 12 weeks with 2-year PFS revealed a trend to statistical significance (*p* = 0.06) in this study. Bhatia et al have demonstrated that a 6-week post-treatment primary tumour absolute volume (> 5.7 cm^3^) and volume reduction (< 35%) threshold could provide > 90% specificity for treatment failure, although with low sensitivity (58% and 26% respectively). Our data also showed that 6-week and 12-week nodal volume was able to predict 2-year PFS. Whilst interval change in nodal volume was not prognostic in this study, previous CT- [32–34] and MRI-based studies have demonstrated a post-treatment percentage reduction in lymph node size to be highly accurate for the identification of residual malignant nodes [18].

Reliable measurements of tumour size are important in order to assess for tumour size and interval change. Although good to excellent reliability of the linear dimensions was achieved in this study (ICC 0.79–0.95), it is known that linear dimensions are prone to measurement error and that the reliability and agreement can be improved by volumetric analysis. Volume measures also have the optimum ability to define change between serial scans when accounting for measurement error. This is corroborated by our finding of 6- and 12-week post-treatment interval changes only being statistically significant on volumetric analysis. However, although algorithms are rapidly evolving, there are currently challenges to the routine use of volume analysis in clinical practice and linear dimensions remain widely practised.

Some limitations of this study are recognised. Firstly, nodal analysis was limited to the single largest node; however, it is possible that this was not representative, and although lymph node cystic and necrotic change analyses were combined, they are known to have differing aetiology and prognostic implications. In addition, the nodal signal evaluation was limited and additional criteria such as nodal DWI signal and T2w signal may be evaluated in future studies [20]. Secondly, the unexpected high proportion of HPV-OPC patients recruited in the prospective study restricted the subgroup analysis, with a limited sample of other HNSCC, and with the low number of treatment failures limiting the interpretation of the comparison with 2-year outcomes.

## Conclusion

Our results would support the premise that 6 weeks post-CRT would be an appropriate interval for a reference MRI following CRT for stage 3 and 4 HNSCC primary tumours. The exceptions are when there is HPV-OPC with nodal disease, or when volumetric analysis is routinely performed, in which case a 12-week post-CRT reference study may be more appropriate.

## Abbreviations

CRT: Chemoradiotherapy
DWI: Diffusion-weighted imaging
HNSCC: Head and neck squamous cell cancer
HPV: Human papillomavirus
IMRT: Intensity-modulated radiotherapy
OPC: Oropharyngeal cancer
PET/CT: Positron emission tomography/computed tomography
PFS: Progression-free survival

## Declarations

### Guarantor

The scientific guarantor of this publication is Dr. S.E.J. Connor.`

### Statistics and biometry

One of the authors has significant statistical expertise.

### Study subjects or cohorts overlap

No study subjects or cohorts have been previously reported at present although overlapping cohorts have been analysed using different imaging data and two studies are under review by the overlapping cohorts with differing imaging data and analysis are currently in press *(Cancer Reports and DentoMaxilloFacial Radiology)*.

### Methodology

• prospective

• cross-sectional study

• performed at one institution
